# Gut microbiota composition is associated with disease severity and host immune responses in COVID-19

**DOI:** 10.3389/fcimb.2023.1274690

**Published:** 2023-12-12

**Authors:** Ruyue Fan, Shuai Liu, Na Sun, Ying Yang, Xia Deng, Bin Hu, Changhua Sun, Chengli Wen, Hui Li, Dong Cheng, Chuanjun Huang, Peibin Hou, Tianliang Zhang

**Affiliations:** ^1^ Shandong Center for Disease Control and Prevention, Jinan, Shandong, China; ^2^ Shandong Provincial Key Laboratory of Infectious Diseases Control and Prevention, Jinan, Shandong, China; ^3^ Department of Respiratory and Critical Care Medicine, Shandong Provincial Hospital Affiliated to Shandong First Medical University, Jinan, Shandong, China; ^4^ Shandong Key Laboratory of Infectious Respiratory Disease, Jinan, Shandong, China; ^5^ School of Public Healthy, Weifang Medical University, Weifang, Shandong, China

**Keywords:** gut microbiota, COVID-19, host immune responses, disease severity, opportunistic pathogens

## Abstract

**Background:**

Human gut microbiota play a crucial role in the immune response of the host to respiratory viral infection. However, evidence regarding the association between the gut microbiome, host immune responses, and disease severity in coronavirus disease 2019 (COVID-19) remains insufficient.

**Methods:**

To better comprehend the interactions between the host and gut microbiota in COVID-19, we conducted 16S rRNA sequencing and characterized the gut microbiome compositions in stool samples from 40 COVID-19 patients and 33 non-pneumonia controls. We assessed several hematological parameters to determine the immune status.

**Results:**

We found that the gut microbial composition was significantly changed in COVID-19 patients, which was characterized by increased opportunistic pathogens and decreased commensal bacteria. The frequency of prevalent opportunistic pathogens *Enterococcus* and *Lactobacillus* increased, especially in severe patients; yet the abundance of butyrate-producing bacteria, *Faecalibacterium*, *Roseburia*, and *Anaerostipes*, decreased significantly, and *Faecalibacterium prausnitzii* might help discriminate severe patients from moderate patients and non-pneumonia people. Furthermore, we then obtained a correlation map between the clinical characteristics of COVID-19 and severity-related gut microbiota. We observed a notable correlation between the abundance of *Enterococcus faecium* and abnormal neutrophil or lymphocyte percentage in all COVID-19 patients. *Faecalibacterium* was positively correlated with lymphocyte counts, while negatively correlated with neutrophil percentage.

**Conclusion:**

These results suggested that the gut microbiome could have a potential function in regulating host immune responses and impacting the severity or consequences of diseases.

## 1 Introduction

The coronavirus disease 2019 (COVID-19), caused by the severe acute respiratory syndrome coronavirus 2 (SARS-CoV-2), is still a health problem worldwide. Patients with COVID-19 can be identified by typical respiratory symptoms; however, common gastrointestinal (GI) symptoms have also been demonstrated to be significant features of the symptomatology associated with COVID-19, such as anorexia, vomiting, and abdominal pain (Zuo et al., 2020; Hayashi et al., 2021). Moreover, gastrointestinal symptoms have been reported to not only appear in patients with COVID-19 but also occur prior to the onset of typical respiratory symptoms (Schmulson et al., 2020). Certain studies note a substantial involvement of the GI tract after SARS-CoV-2 infection, such as the capability of the virus to infect and replicate in human small intestine enterocytes (Lamers et al., 2020), the positive detection of surviving virus particles in stool samples (Merola et al., 2020), and gut microbiota dysbiosis occurring in patients with COVID-19 (Zuo et al., 2020). Previous research has indicated that SARS-CoV-2 enters the human body via the angiotensin-converting enzyme 2 (ACE2) receptor, which has significant expression in epithelial cells of the lung and intestine (Lucas et al., 2020). Given the high distribution of ACE2 receptors in the intestinal mucosa, the GI tract becomes a crucial biological niche for the expansion of pathogens into the respiratory and cardiovascular systems.

The dysregulated inflammatory response of the host is the key to determining disease severity and progression (Lucas et al., 2020; Cao et al., 2021). When the virus enters a cell, it has the potential to trigger innate and adaptive host immune responses, which are essential parts of the defense against viral infections (Schultze and Aschenbrenner, 2021; Sette and Crotty, 2021). The dysregulated inflammatory responses could contribute to tissue degradation and the development of the disease (Nasrollahi et al., 2023). These include the overproduction of inflammatory cytokines, overactivation of neutrophils and macrophages, T lymphopenia, and dysregulated T- and B-lymphocyte function, when there is preexisting immunity to the virus (Wilk et al., 2020; Schultze and Aschenbrenner, 2021; Sette and Crotty, 2021). In the gut, the local immune disorders were closely associated with gut bacterial dysbiosis and instability, which further exacerbated the impairment of gut health.

Human intestinal microbial communities play a crucial role in maintaining host homeostasis through various mechanisms such as immunomodulation, nutrient metabolism, and structural protection against pathogens. The GI tract is recognized as the major immunological organ, and its resident microbiota are known to be involved in modulating the host immune system and our entire physiology (Donaldson et al., 2016; Schirmer et al., 2016). Respiratory viral infection has been found to result in notable alterations in the gut microbiome, even in the absence of identifiable virions inside the GI tract (Lu et al., 2017; Hanada et al., 2018; Libertucci and Young, 2019). Several studies also described a possible connection between COVID-19 severity and gut microbiota composition (Dhar and Mohanty, 2020; Yeoh et al., 2021; Zuo et al., 2021). The relative abundance of *Clostridium innocuum*, *Alistipes finegoldii*, and *Ruthenibacterium lactatiformans* was positively correlated with inflammatory biomarkers and tended to increase with COVID-19 progression (Schult et al., 2022). COVID-19 severity was correlated with an increased abundance of *Bacteroides* but a reduced abundance of *Roseburia* (Hazan et al., 2022). Additionally, a negative correlation between disease severity and the abundance of bacteria associated with tryptophan metabolism and maintenance of immune homeostasis was observed in COVID-19 patients (Reinold et al., 2021). Nevertheless, data revealing the holistic interplay between the gut microbiota and host immune responses to SARS-CoV-2 infection still remain limited. There is still substantial work to be done to address the direct causality between gut microbiota and COVID-19 deterioration or prognosis.

Here, we conducted a characterization of the gut microbiome and plasma inflammatory cytokines among 18 moderate and 22 severe patients with COVID-19 during hospitalization and 33 individuals with no symptoms or recovery from mild infection. Furthermore, we analyzed the diversity and composition of gut microbiota and uncovered the possible links between gut microbiota and host systemic immune responses. This will provide valuable insight into the function of the gut microbiota and shed light on the development of gut microbiota-based treatment for COVID-19.

## 2 Materials and methods

### 2.1 Subject recruitment and sample collection

Hospitalized patients with COVID-19 were recruited during the peak SARS-CoV-2 season of 2022–2023 in the Department of Pulmonary and Critical Care Medicine of Shandong Provincial Hospital. All patients were diagnosed with pneumonia by chest computed tomography scans. The throat swab samples were tested by reversing real-time polymerase chain reaction (RT-PCR) to detect the presence of SARS-CoV-2. Referring to the New Coronavirus Pneumonia Diagnosis and Treatment Program (10th edition) published by the National Health Commission of China, severe pneumonia was defined as dyspnea, respiratory frequency ≥30/min, blood oxygen saturation ≤93%, partial pressure of arterial oxygen (PaO_2_)-to-fraction of inspired oxygen (FiO_2_) ratio ≤300, and/or lung infiltrates >50% within 24–48 h, respiratory failure, septic shock, and/or multiple organ dysfunction or failure. Moderate pneumonia was defined as fever or respiratory tract infection symptoms with imaging indicating pneumonia. Patients were classified as “non-pneumonia” if there were no radiographic indications of pneumonia with minor clinical symptoms. Blood and stool samples from in-hospital patients were collected, flash-frozen, and stored in liquid nitrogen at −80°C until use.

### 2.2 Stool DNA extraction, 16S rRNA sequencing, and data processing

A total of 73 stool samples were collected from COVID-19 patients at admission and donors recovering from mild infections or asymptomatic individuals. All samples were stored at −80°C immediately until processing. According to the manufacturer’s instructions for the QIAamp Fast DNA Stool Mini Kit (Qiagen, Germany), stool DNA was extracted. Approximately 200 mg of stool samples was used for DNA extraction and sequencing libraries. PCR was performed to amplify the V3–V4 hypervariable region of the 16S rRNA gene in all samples, following the guidelines provided in the 16S Metagenomic Sequencing Library Preparation guide. The amplicon primers were as follows: forward primer, 5′-AGAGTTTGATCCTGGCTCAG-3′; and reverse primer, 5′-GNTACCTTGTTACGACTT-3′. The PCR conditions were as follows: initial denaturation at 95°C for 3 min; 30 cycles of denaturation at 95°C for 30 s, annealing at 56°C for 30 s, extension at 72°C for 3 min; at 72°C for 10 min; hold at 4°C. The PCR products that had been purified were combined in equal numbers and subsequently utilized to generate the amplicon library for single-molecule real-time (SMRT) sequencing analysis using the PacBio sequencing platform (Novogene Co., Ltd., Beijing, China). The reads obtained from the platform were subjected to processing using SMRT software to eliminate the unqualified reads (including excessively long or short reads, chimeras, and inferior-quality reads). Subsequently, qualified sequences were clustered and divided into operational taxonomic units (OTUs) based on a threshold of 97% similarity, as described by Liu et al. (Zhang et al., 2021). Raw sequence data generated for this study are available in the Sequence Read Archive under BioProject accession PRJNA1004732.

### 2.3 Community structure and diversity analysis

Sequence analysis was conducted using Uparse software (Uparse v7.0.1001) (Edgar, 2013). Sequences with ≥97% similarity were allocated to identical OTUs. The representative sequence of each OTU was subjected to a screening process for further annotation. For every representative sequence, the SSUrRNA Database of the Silva Database was used based on the Mothur algorithm to annotate taxonomic information (Quast et al., 2013). Prior to analyzing alpha-diversity and beta-diversity, the original OTU table was rarefied to 5,599 reads (the smallest number of bacterial reads among samples) to allow for comparable comparisons. The alpha-diversity indices, including observed species, Chao1, Shannon, Simpson, and ACE, were determined by rarefaction analysis using Mothur software (version 1.21.1). The indices in our samples were computed using QIIME (version 1.9.1) and visualized using R software (version 2.15.3). Beta-diversity analysis was used to assess variations of samples in species complexity, and the QIIME software (version 1.9.1) was utilized to quantify beta-diversity on both weighted and unweighted UniFrac (Lozupone et al., 2011). Principal coordinate analysis (PCoA) and analysis of similarities (ANOSIM) based on the Bray–Curtis similarity were used to assess the statistical significance of the diversity index variation among the samples. The Kruskal–Wallis rank-sum test was used to examine the alterations and disparities between samples. The construction of PCoA was performed using the WGCNA package, stat packages, and ggplot2 package in R software (version 2.15.3). A *p-*value <0.05 was considered significant, and 0.05 ≤ *p* < 0.10 was considered a tendency. Linear discriminant analysis (LDA) effect size (LEfSe) analysis was performed to define COVID-19-related microbial characteristics.

### 2.4 Correlation between gut microbes and clinical features of the host

Tests for blood cells, inflammation biomarkers, and albumin of COVID-19 patients were performed using an automated hematology and biochemical analyzer. The full whole blood cell subsets were analyzed using the CytoFLEX S Flow Cytometer (Beckman Coulter, Brea, CA, USA) with FlowJo V10.7.2 software. A correlation matrix between gut microbes from the moderate and severe patients and their clinical features of host values was then generated using Spearman’s rank correlation coefficient implemented in R version 4.2.3. Spearman’s correlations were calculated using the function corr.test of the R package psych, and the correlation heatmap was drowned using the R package ggplot2. Student’s *t*-test was performed to determine the significance analysis of the means between groups. *p < 0.05, **p < 0.01, and ***p < 0.001 represent significant differences.

## 3 Results

### 3.1 Clinical cohorts

COVID-19 patients were recruited between 2022 and 2023 (*n* = 40). Out of our cohort of 73 subjects, 18 were classified as “moderate”, 22 as “severe”, and 33 as “non-pneumonia” based on the criteria outlined in the Materials and Methods section. The three groups were comparable in terms of sex. All COVID-19 patients enrolled had at least one comorbidity, with pneumonia disease being the most prevalent. The superior respiratory support strategy was chosen and summarized. Among all patients, 85% of patients (34 of 40) needed supplemental oxygen. In the severe group, 40.91% of patients (9 of 22) required superior respiratory support (such as mechanical ventilatory support). The hospital mortality rate in the severe cases of pneumonitis was 9.09% (2 of 22). The chest computed tomography from severe patients who eventually progressed to death showed confluent and predominantly patchy ground-glass opacities with pronounced peripheral distribution and partial consolidation (**Supplementary Figures 1A,B**). The severe group had a significantly higher percentage of neutrophil counts and C-reactive protein (CRP) and human interleukin (IL)-6 levels whereas substantially reduced lymphocyte counts and PaO_2_/FiO_2_ ratio levels compared to the moderate group. The characteristics of cohorts are listed in **Table 1**.

**Table 1:** Demographics of the COVID-19 and non-pneumonia cohorts.

	Non-pneumonia (N = 33)	Moderate (N = 18)	Severe (N = 22)	*p*-Value
Gender				0.0754
Male	12 (36.36%)	12 (66.66%)	13 (59.09%)	
Female	21 (63.63%)	6 (33.33%)	9 (40.90%)	
Age, years (mean (SD))	71.67 (18.34)	66.06 (14.79)	69.95 (14.87)	0.5134
Comorbidity				
Pneumonia disease	0 (0%)	18 (100%)	22 (100%)	–
Cancer	0 (0%)	4 (22.22%)	1 (4.54%)	0.0097
Hypertension	4 (12.12%)	7 (38.89%)	9 (40.91%)	0.0289
Diabetes	1 (3.03%)	5 (27.78%)	7 (31.82%)	0.0106
Coronary heart disease	4 (12.12%)	7 (38.89%)	7 (31.82%)	0.0685
Cerebrovascular disease	3 (9.09%)	3 (16.67%)	4 (18.18%)	0.5767
Laboratory tests (mean (SD))				
Total leukocytes (×10^9^/L)	NA	7.89 (4.20)	8.29 (3.28)	0.7369
Neutrophil (×10^9^/L)	NA	5.81 (3.77)	6.68 (2.98)	0.4208
Proportion of neutrophils (%)	NA	70.38 (13.24)	79.32 (10.53)	0.0225
Lymphocytes (×10^9^/L)	NA	1.52 (1.03)	0.97 (0.64)	0.0452
Proportion of lymphocytes (%)	NA	25.31 (17.73)	13.19 (8.53)	0.0073
Platelet (×10^9^/L)	NA	216.72 (70.36)	228.41 (104.41)	0.6877
C-reactive protein (mg/l)	NA	11.46 (14.63)	28.72 (38.30)	0.0810
Procalcitonin (ng/ml)	NA	0.11 (0.09)	0.16 (0.17)	0.2462
Interleukin-6 (pg/ml)	NA	8.32 (9.68)	27.15 (30.63)	0.0198
PaO_2_/FiO_2_ grade (mmHg)	NA	434.99 (77.66)	221.10 (75.99)	0.0000
>300	NA	18 (100%)	2 (9.10%)	0.0000
>200 to ≤300	NA	0 (0%)	11 (50.00%)	0.0004
>100 to ≤200	NA	0 (0%)	8 (36.36%)	0.0042
≤100	NA	0 (0%)	1 (4.55%)	0.3596
Respiratory support				
Non-invasive mechanical ventilatory support	NA	0 (0%)	2 (9.09%)	0.1894
High-flow nasal cannula oxygen therapy	NA	0 (0%)	7 (31.82%)	0.0084
Low-flow oxygen therapy	NA	12 (66.67%)	13 (59.09%)	0.6225
Outcome				
Admission to ICU	NA	1 (5.56%)	3 (13.64%)	0.3725
Death	NA	0 (0%)	2 (9.09%)	0.1894

Note. Data are mean (SD) or n (%). p-Values were calculated using Student’s t-test, χ^2^ test, or Fisher’s exact test, as appropriate.

NA, not applicable; SD, standard deviation; COVID-19, coronavirus disease 2019; PaO_2_/FiO_2_, partial pressure of arterial oxygen to fraction of inspired oxygen; ICU, intensive care unit; COVID-19, coronavirus disease 2019.

### 3.2 Alteration of gut microbiota composition in patients with COVID-19

**Figure 1:**
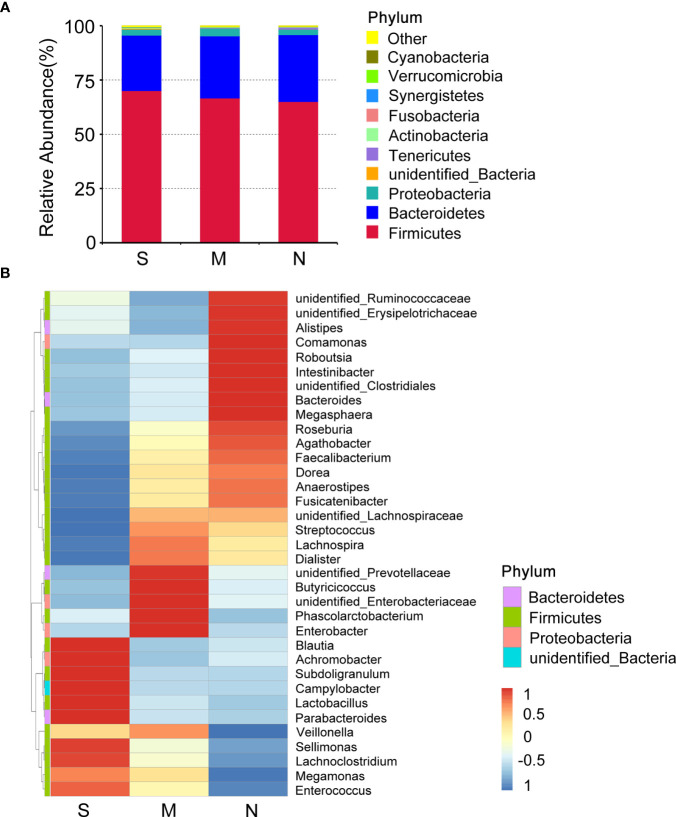
Composition differences in gut microbiota between coronavirus disease 2019 (COVID-19) patients and non-pneumonia individuals. **(A)** Top 10 phyla in stool samples from patients with severe and moderate COVID-19 and non-pneumonia individuals. The dominant phyla were *Firmicutes* and *Bacteroidetes* in all groups. The x-axis indicates group names (S: severe; M, moderate; N, non-pneumonia individuals). The y-axis indicates relative abundance. “*Others*” indicates the sum of the relative abundance of all phyla except the top 10. “*unidentified_Bacteria*” represents OTUs that could not be assigned at the phylum level. **(B)** Heatmap of the relative abundance of the top 35 most abundant genera among the three groups. The values corresponding to the heatmap are Z-values obtained by standardizing the relative abundance of the species in each row. The hierarchical taxon clustering on the left was based on the Euclidian distance. The left vertical bars correspond to the phylum level classification of each genus; the top 35 genera belonged to three phyla. *Blautia* is dominant in the severe groups.

**Figure 2:**
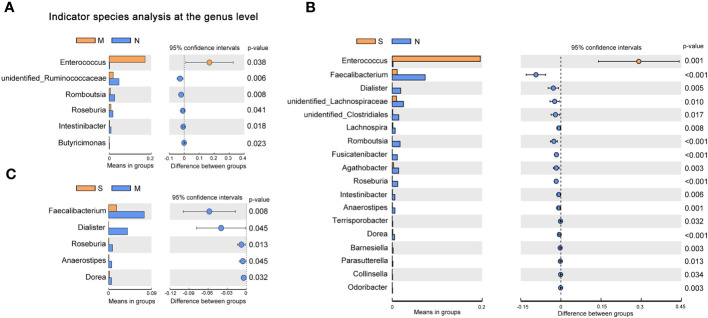
Changes of gut microbiota composition in the patients with coronavirus disease 2019 (COVID-19). Differentially bacterial species among the patients with severe, moderate COVID-19, and non-pneumonia individuals. operational taxonomic units (OTUs) and taxon differences are shown with *p*-values less than 0.05. **(A)** The differences between the moderate and non-pneumonia groups. **(B)** The differences between the severe and non-pneumonia groups. **(C)** The differences between the moderate and severe groups.

To investigate the distribution of bacterial taxa, the composition of the gut microbes at the phylum level was subjected to visualization. The results showed that in patients and controls, *Firmicutes*, *Bacteroidetes*, and *Proteobacteria* were dominant communities. Particularly, the phylum Tenericutes was relatively more abundant in severe COVID-19 patients than in non-pneumonia individuals (mean <0.01% *vs.* 0.8%; *p* < 0.01, Mann–Whitney test) (**Figure 1A**, **Supplementary Figure 2A**). The corresponding heatmap of the top 35 abundant genera is shown in **Figure 1B** and **Supplementary Figure 2B**. From these results, it was found that most of the members of the top 35 genera were classified as phyla *Firmicutes*, *Bacteroidetes*, and *Proteobacteria*. Moreover, the dominant genera were different between samples; *Blautia* was dominant in the severe groups, while *unidentified* Prevotellaceae dominated in the moderate group. The differences of microbial communities at the genus level were further analyzed, and the higher enrichment of *Enterococcus* was observed in the COVID-19 patients group compared with the control group (M *vs.* N, *p* = 0.038; S *vs.* N, *p* = 0.001) (**Figures 2A, B**). Moreover, 4 and 15 bacterial genera (e.g., *Romboutsia* and *Roseburia*) were abundant in the moderate and severe COVID-19 groups, and 15 bacterial genera (e.g., *Faecalibacterium* and *Dialister*) were decreased in the severe COVID-19 group compared with the control group. In addition, the significant depletion of the genera *Faecalibacterium*, *Dialister*, and *Roseburia* was found in the severe COVID-19 patients compared with the moderate group (**Figure 2C**). To better identify bacteria with value as potential COVID-19 indicators, bacterial communities at the species level were evaluated. The abundance of several bacteria showed obvious differences between the COVID-19 patients and non-pneumonia groups (**Supplementary Figures 3A,B**). Particularly, *Enterococcus faecium* was enriched in the severe group (S *vs.* N, *p* = 0.001), while *Bacteroides plebeius* and *Clostridium celatum* were decreased compared with the control group (**Supplementary Figure 3B**). Furthermore, the abundance of four bacterial species, including *Faecalibacterium prausnitzii*, *Anaerostipes hadrus*, *Roseburia faecis*, and *Coprococcus comes*, was obviously lower in the severe group compared to the moderate group (**Supplementary Figure 3C**). These results demonstrated that *Enterococcus* was significantly elevated in the COVID-19 group, which indicated that *Enterococcus* had an obvious effect in distinguishing COVID-19 and non-pneumonia groups and could be used as a potential candidate for aiding in the diagnosis and prediction of the disease severity of COVID-19.

### 3.3 Bacterial community diversity in patients with COVID-19

**Figure 3:**
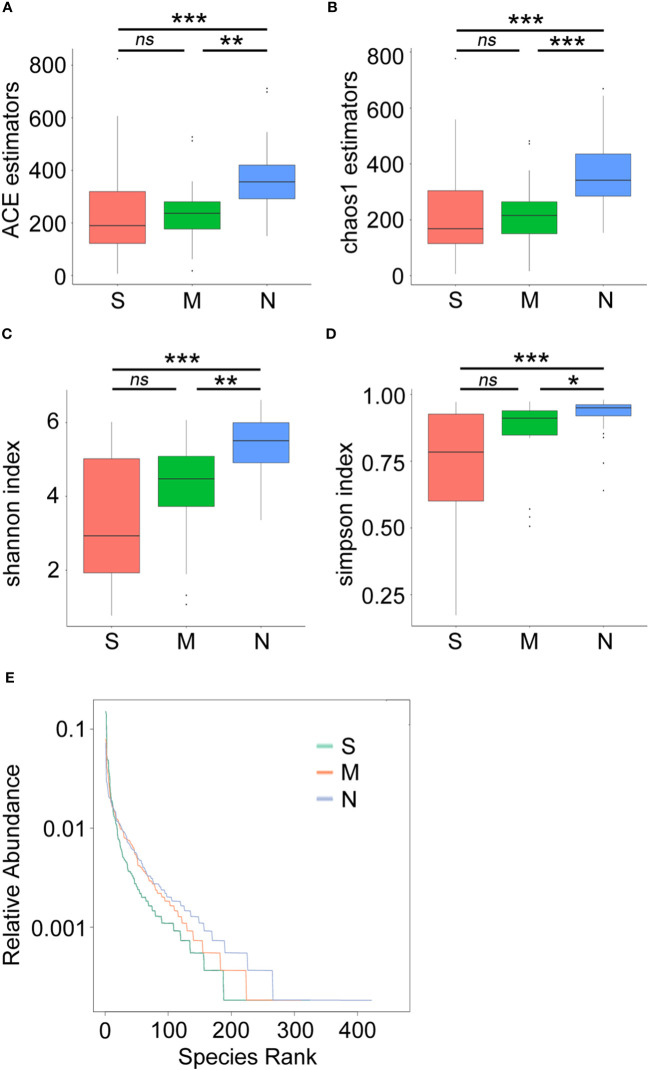
Alpha-diversity analysis of microbial community composition in stool samples from patients with coronavirus disease 2019 (COVID-19) and non-pneumonia individuals. The boxplots represent the minimum, maximum, and median of the sample values obtained from the richness estimators ACE **(A)** and Chao1 **(B)** and diversity indices Shannon **(C)** and Simpson **(D)**. The rank abundance curve represents richness of bacterial community **(E)** *p < 0.05, **p < 0.01, ***p < 0.001, ns, not significant.

**Figure 4:**
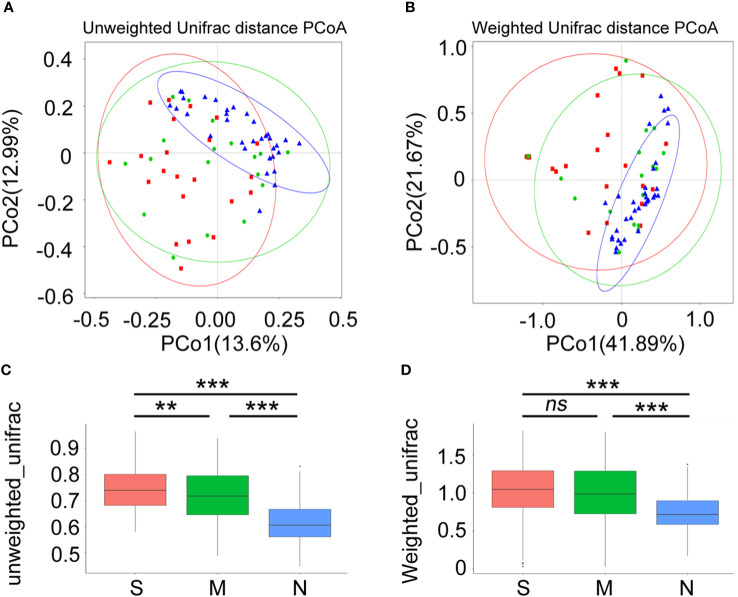
Beta-diversity analysis of gut microbiota composition of coronavirus disease 2019 (COVID-19) patients and non-pneumonia individuals. Principal coordinate analysis (PCoA) of gut microbiota based on unweighted UniFrac distance **(A)** and weighted UniFrac distance **(B)** in three groups. Blue dots refer to the non-pneumonia group. Red and blue dots refer to the severe and moderate groups, respectively. The ellipses represent the 68% confidence interval for each time point. The samples from COVID-19 patients and non-pneumonia individuals showed clustering distributions. **(C, D)** The boxplots show the extent of gut microbiota compositional changes among patients with severe, moderate COVID-19 and non-pneumonia individuals. **p < 0.01, ***p < 0.001, ns, not significant.

Next, a diversity analysis was performed to gain a deeper understanding of the species richness and microbiome composition of each group. It was found that compared with the non-pneumonia group, the alpha-diversity indices (ACE, Chao1, Shannon, and Simpson) were significantly reduced in the moderate and severe groups (*p* < 0.05). However, no significant difference was observed between the moderate and severe patients (**Figures 3A–D**). This indicated that the uniformity of bacterial community was lower in COVID-19 cases than in non-pneumonia cases but was similar in the moderate and severe groups. The rank abundance curve reflected the similarity in structure across all microbial communities, as seen by the same patterns observed in both the frequency and abundance of grades within the same group of samples, and the control group had higher species richness and uniformity than the COVID-19 groups (**Figure 3E**). To further demonstrate microbiome space among the three groups, the beta-diversity analysis was performed. PCoA was used to analyze two classic beta-diversity indices, and the separate clusters of the COVID-19 and control groups at the species level were confirmed. The PCoA plot, which was generated using unweighted UniFrac distances, revealed a significant difference between the non-pneumonia group and the other two groups, and PCo1 and PCo2 accounted for 13.6% and 12.99% of the total analysis results, respectively (**Figure 4A**). Similarly, weighted UniFrac distance-based bacterial structure analysis showed that the samples in the non-pneumonia group were discriminated against those in the moderate and severe groups. PCo1 and PCo2 explained 41.89% and 21.67% of the variation, respectively (**Figure 4B**). These results indicated different bacterial communities in the COVID-19 groups compared to the non-pneumonia group. The significant difference between the two indices (unweighted and weighted UniFrac distances) among the three groups was shown using the Wilcoxon rank-sum test (**Figures 4C, D**). According to the distance index ranking, ANOSIM provided confirmation that the distance between groups was considerably larger than the distance within groups, demonstrating a major difference in the microbiome structure across various groups (**Supplementary Figures 4A–C**).

### 3.4 Gut microbiota with significant differences in groups

**Figure 5:**
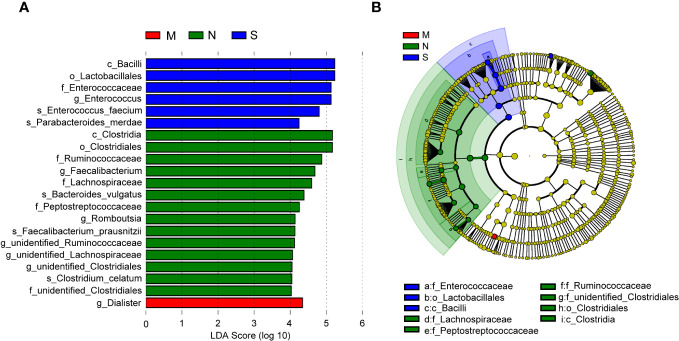
Linear discriminant analysis effect size (LEfSe) analysis of bacterial species. **(A)** The bar graph shows the linear discriminant analysis (LDA) scores calculated for characteristics at the operational taxonomic unit (OTU) levels. Blue and red bars refer to the severe and moderate groups, respectively. The red ones refer to the non-pneumonia group. **(B)** The cladogram shows the relative abundance of OTUs. Green and blue (light and dark green or blue) areas represent the non-pneumonia and severe groups, respectively. Green, blue, and red nodes in the branches represent bacterial species that played an important role in their groups. Yellow nodes represent bacterial species that did not play an important role in both groups.

In order to identify bacterial taxa (at taxonomic levels) that were differentially abundant among the three groups, we used LEfSe analysis, at an LDA score >4. The results showed that the family Enterococcaceae, genus *Enterococcus*, and two species (*Parabacteroides merdae* and *E. faecium*) were abundant in the severe COVID-19 group, while the genus *Dialister* was enriched in the moderate group (**Figure 5A**). In the non-pneumonia group, three domain families (Lachnospiraceae, Peptostreptococcaceae, and Ruminococcaceae) and two genera (*Faecalibacterium* and *Romboutsia*) were detected. In addition, the cladogram analysis of taxa also showed a significant difference in gut microbiota among the three groups, and the severe group was characterized by taxa in the family Enterococcaceae (**Figure 5B**). Of note is that the two severe cases eventually progressed to death, and the abundance of *Enterococcus* and Lactobacillales accounted for 87% and 54% of their gut microbiota, respectively (**Supplementary Figures 1C,D**).

### 3.5 Associations between gut microbial species and specific biomarkers or clinical parameters

**Figure 6:**
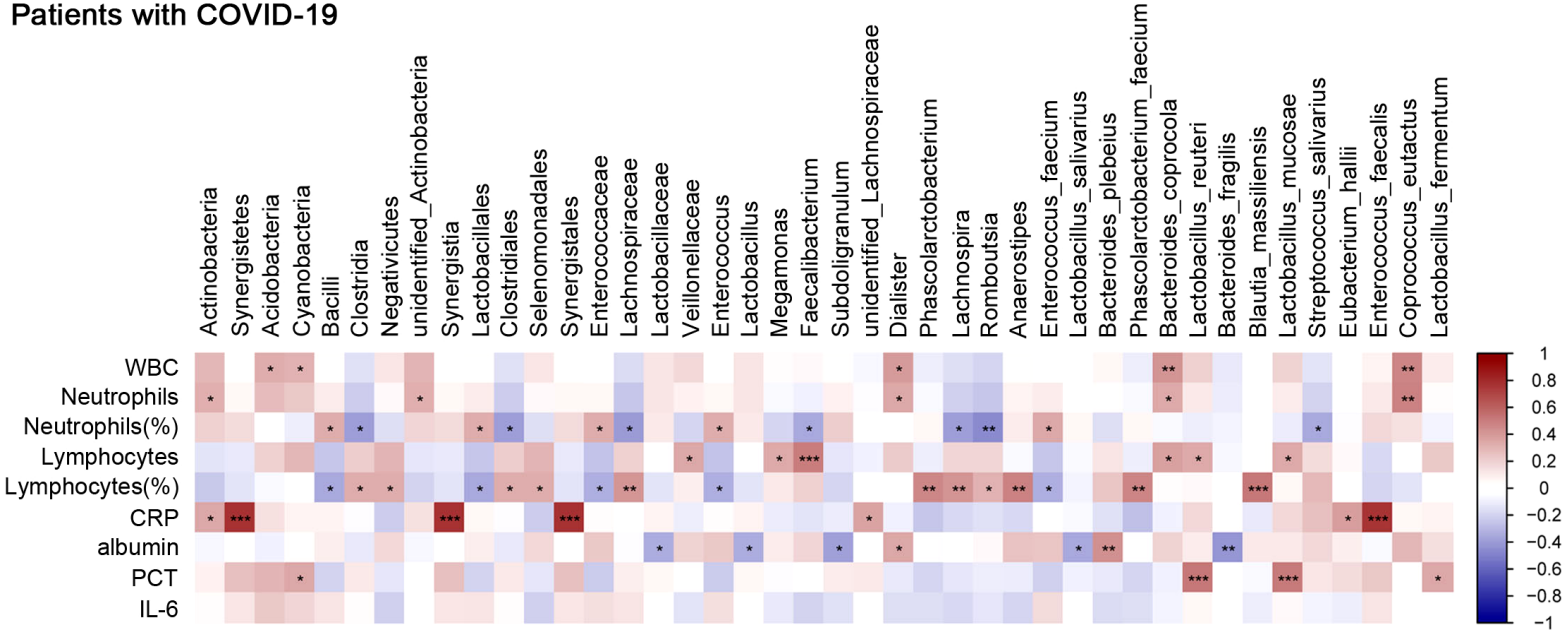
Correlation between gut microbes and clinical parameters that reflect host immune response. Analysis of the correlation between gut microbes and clinical traits. Red bars indicate positive associations, while blue bars indicate negative associations. The color key indicates the association strength and direction in terms of the *t*-test. Pearson’s correlation coefficient and *p*-value are used for plotting. Black asterisks indicate associations with *p*-value. *p < 0.05, **p < 0.01, and ***p < 0.001 represent significant differences.

To explore the interplay of the gut microbiota with certain specific blood biomarkers or clinical parameters that reflect the host immune response, we first investigated the distribution of bacterial tax in all COVID-19 patients. The results showed that at the phylum level, *Firmicutes* and *Bacteroidetes* were dominant communities (**Supplementary Figure 5A**). We further assessed the correlation between specific species enriched or depleted in COVID-19 patients and inflammatory indicators. The results are listed in **Supplementary Table 1**. At the species level, we found that *Phascolarctobacterium faecium*, *Bacteroides coprocola*, *Lactobacillus reuteri*, *Blautia massiliensis*, and *Lactobacillus mucosae* were positively correlated with lymphocyte counts or lymphocyte percentage, while *E. faecium* was negatively correlated with lymphocyte percentage, which suggested a potential supplementary function of these bacteria in the lymphoid reactions to SARS-CoV-2 infection. *E. faecium*, *B. coprocola*, and *Coprococcus eutactus* were positively correlated with neutrophil percentage or neutrophil counts, while *Streptococcus salivarius* was negatively correlated with neutrophil percentage (**Figure 6**), which suggested that these bacteria were closely involved in neutrophil-mediated inflammatory responses. Moreover, *Eubacterium hallii* and *Enterococcus faecalis* were positively correlated with CRP; *L. reuteri*, *L. mucosae*, and *Lactobacillus fermentum* were positively correlated with procalcitonin (PCT). Due to many COVID‐19 patients developing hypoalbuminemia at admission, we further analyzed the correlation between gut microbes and albumin. *B. plebeius* was found to be positively correlated with albumin, while *Lactobacillus salivarius* and *Bacteroides fragilis* were inversely related to albumin, indicating a potential connection between gut microbes and potential organ injury in COVID-19 patients. We used LEfSe analysis, at an LDA score >4. The results showed that the genus *Faecalibacterium* was abundant in the moderate COVID-19 group, while species *E. faecium* was enriched in the severe group (**Supplementary Figure 5B**). We also selected and analyzed some differentially abundant gut microbiota between two groups in LDA. At the genus level, *Faecalibacterium* was positively correlated with lymphocyte counts while negatively correlated with neutrophil percentage. We further evaluated the association with immunological indicators at the species level and found that *E. faecium* was positively correlated with neutrophil percentage while negatively correlated with lymphocyte percentage (**Supplementary Figure 5C**). Overall, these data suggested an association between gut microbiota composition and aberrant immune responses.

## 4 Discussion

Since late 2019, the SARS-CoV-2 strains, including the Delta and Omicron mutation types, have caused complicated multisystem diseases. In addition to different initial respiratory symptoms, gastrointestinal symptoms, including diarrhea, nausea, and vomiting, are common clinical manifestations. The ACE2 receptor modulates innate immunity and affects gut microbiota composition (Perlot and Penninger, 2013). SARS-CoV-2 infection is initiated in human intestinal epithelial cells by the binding of the viral spike protein to ACE2, thereby disrupting intestinal immune homeostasis in the host and resulting in the adverse consequences of COVID-19. Growing evidence suggests that the crosstalk between lung immunity and gut microbiota, which is called the gut–lung axis, is actively involved in the protective and pathologic immune responses to SARS-CoV-2 (de Oliveira et al., 2021; Rastogi et al., 2022). Therefore, changes in intestinal microbiota communities may significantly affect disease severity and progression as well as post-COVID-19 syndrome development (Mahooti et al., 2020; Ancona et al., 2023).

A previous study on the gut microbiota of hospitalized patients with COVID-19 revealed significant changes in microbiota communities, with an increased presence of opportunistic microorganisms and a decrease in beneficial microbes (Zuo et al., 2020). Studies have reported that after SARS-CoV-2 infection, the abundance of *Faecalibacterium*, *Coprococcus*, *Ruminococcus*, and *Roseburia* was decreased (Tao et al., 2020; Gaibani et al., 2021), whereas that of *Enterococcus*, *Rothia*, and *Lactobacillus* was increased (Tao et al., 2020; Wu et al., 2021). In the present study, we observed that the microbial relative abundance at the taxonomic level was associated with COVID-19 severity. Furthermore, bacterial diversity was decreased in the gut microbiota of patients with COVID-19, particularly in the severe group. Although we observed no significant differences in alpha-diversity between the moderate and severe groups, there were obvious changes in bacterial composition. This finding may be attributed to similar comorbidities or antibiotic usage between both cohorts. Moreover, we observed a consistent decrease in the alpha-diversity of the gut microbiota of patients with COVID-19 compared with that of the healthy population. This can be attributed to direct infection and the indirect effects of SARS-CoV-2. First, healthy gut microbiota may be vital for balancing the optimal immune responses against harmful excessive inflammatory responses. However, SARS-CoV-2 directly binds to ACE2 in intestinal epithelial cells and induces gut microbiota imbalance, further decreasing floral diversity in the intestine and the abundance of short-chain fatty acid-producing bacteria and increasing the abundance of opportunistic bacteria belonging to the family Enterobacteriaceae. Second, SARS-CoV-2 infection can affect the gut–lung axis and alter the functional and metabolic characteristics of the gut microbiota, further changing the abundance and diversity of the gut microbiota in the gastrointestinal tract (Zhang et al., 2023).

Notably, in the present study, the abundance of *Enterococcus* was increased in the severe group compared with the moderate and non-pneumonia groups. *Enterococcus* is a typical opportunistic pathogen. Some studies have revealed that enterococci may be associated with the development of severe illnesses. For example, *Enterococcus* may contribute to the development of cytokine storms in some patients with COVID-19 by activating Toll-like receptors (Gago et al., 2022; Righi et al., 2022). Furthermore, the excessive proliferation of *Enterococcus* leads to subsequent intestinal dysbiosis, intestinal epithelial barrier injury, and secondary infections (Bäumler and Sperandio, 2016; Hanada et al., 2018). *E. faecium* is an anaerobic opportunistic pathogen with extensive antibiotic resistance. It often causes severe healthcare-related infections, particularly in critically ill patients admitted to the intensive care unit (Fiore et al., 2019). In the present study, compared with the non-pneumonia group, the abundance of *E. faecium* was increased in the severe group. Moreover, there was a correlation between *E. faecium* and neutrophil or lymphocyte percentage in all patients with COVID-19. Collectively, these results suggest that *Enterococcus* has an obvious effect on distinguishing the COVID-19 and non-pneumonia groups and can be used as a potential candidate for diagnosing and predicting the severity of COVID-19.

*Lactobacillus* can produce lactic acid to regulate immunity and maintain intestinal barrier function (Hou et al., 2018; Wu et al., 2020). Tang et al. have reported that the abundance of *Lactobacillus* is below or close to the lower limit of the normal range in critically ill patients (Tang et al., 2020). Furthermore, another study has reported that hypoalbuminemia is associated with COVID-19 outcomes (Huang et al., 2020). This can be owing to increased capillary permeability resulting from a systemic inflammatory response, leading to albumin leakage into the interstitial space, poor nutritional status, and impaired liver function. In the present study, we observed that the relative abundance of *Lactobacillus* was dominant in severe patients and negatively correlated with albumin levels in patients with COVID-19; this suggests that *Lactobacillus* is associated with liver function maintenance. Furthermore, the abundance of *L. reuteri*, *L. mucosae*, and *L. fermentum* was positively correlated with the abnormal PCT concentration in the plasma of patients with COVID-19. The increase in *Lactobacillus* may exacerbate inflammatory responses against viral pneumonia and is positively correlated with disease severity. Therefore, additional studies are warranted to determine the dynamics of beneficial intestinal bacteria under the stress of viral infections.

Of note, in patients with severe COVID-19 who eventually died, we observed that the relative abundance of *Lactobacillus* and *Enterococcus* was dominant; furthermore, the percentage of neutrophils was higher and that of lymphocytes was lower. Lung lobe-based analysis revealed the typical imaging features of multiple ground-glass opacities with consolidation, which may be attributed to the ability of the intestinal microbiota to affect lung disease progression via the gut–lung axis. Many studies have suggested that gut dysbiosis plays a vital role in the pathogenesis of sepsis and acute respiratory distress syndrome (ARDS) (Chakraborty et al., 2022). The decrease in microbial diversity and accumulation of opportunistic microorganisms can lead to dysbiosis; this, in turn, may lead to immune dysregulation and even ARDS, similar to the manifestations observed in severe COVID-19 (Trompette et al., 2014; Anand and Mande, 2018). Therefore, additional studies on the relationship among inflammatory responses, gut microbiota, and COVID-19 severity are warranted.

Butyrate plays an important role in inhibiting the growth of opportunistic pathogens, maintaining the integration of the intestinal barrier, and activating or enhancing adaptive immune responses (Louis and Flint, 2009; Park et al., 2015). Studies have confirmed that the enrichment of the butyrate-producing genus *Faecalibacterium* is negatively correlated with disease severity (Li et al., 2021; Yeoh et al., 2021) and that the abundance of *F. prausnitzii*, which favors an anti-inflammatory microenvironment, is inversely correlated with COVID-19 severity (Zuo et al., 2020). Our study results are consistent with these findings. We observed that the abundance of *Faecalibacterium*, *Roseburia*, and *Anaerostipes* was decreased in patients with severe COVID-19. Of note, butyrate-producing bacteria such as *Faecalibacterium*, *Anaerostipes*, and Lachnospiraceae were positively correlated with several lymphoid-related markers, including lymphocyte count and lymphocyte percentage; this indicates a potential interaction between the gut microbiota and lymphocyte regulation. Indeed, previous studies have demonstrated that butyrate or butyrate-producing bacteria can modulate the quantity and function of regulatory T cells while also activating T helper cells (Park et al., 2015; Zhu et al., 2018). Therefore, the decrease in butyrate-producing bacteria may exacerbate inflammatory responses against viral pneumonia and exhibit a positive correlation with disease severity. We inferred that the changes in the abundance of *Faecalibacterium* can help accurately distinguish between critical and general patients and that microbial characterization and classification may be powerful tools to predict disease severity.

The present study has some limitations. First, we did not conduct metabolomic, transcriptomic, or proteomic studies or analyze their relationship with changes in the gut microbiota. Determining how gut bacteria affect host intestinal immune responses through microbial metabolites is vital. Second, several factors, including diet, may affect the human gut microbiota. However, data on lifestyle and diet were lacking; nevertheless, all patients had a similar diet, at least during hospitalization, and their meals were managed by the hospital. Third, stool samples were obtained from the patients with COVID-19 after antibiotic treatment and short-term hormone therapy; this may have affected the intestinal bacteria. Fortunately, all patients received the same class of antibiotics (moxifloxacin or third-generation cephalosporin). Therefore, species comparison between the moderate and severe groups was conducted under the same conditions.

## 5 Future perspectives

Only one stool sample was collected from each patient within 24 h of admission, and the dynamic changes in gut microbiota were not elucidated. Incorporating these dynamic data of patients after discharge and expanding the clinical information of the non-pneumonia cohort may provide additional power to our study. Furthermore, future studies with larger sample sizes and more advanced techniques are warranted to confirm these findings and investigate the mechanisms underlying the observed associations.

## 6 Conclusion

In the present study, we used 16S rRNA sequencing to characterize the gut microbiome compositions. The gut microbial composition was significantly changed in COVID-19 patients, and the opportunistic pathogens were increased, yet the commensal bacteria were decreased. Furthermore, a correlation map between clinical hematological parameters and gut microbiota was obtained from COVID-19 patients, demonstrating that gut microbiota could have a function in regulating host immune responses and possibly impacting the severity and consequences of diseases. Still, more in-depth research is warranted to understand the immune-related pathogenesis of COVID-19 in the future.

## Data availability statement

The data presented in the study are deposited in the NCBI repository, accession number PRJNA1004732.

## Ethics statement

The studies involving humans were approved by Shandong Provincial Hospital Ethics Committee. The studies were conducted in accordance with the local legislation and institutional requirements. Written informed consent for participation in this study was provided by the participants’ legal guardians/next of kin. Written informed consent was obtained from the individual(s) for the publication of any potentially identifiable images or data included in this article.

## Author contributions

RF: Conceptualization, Data curation, Funding acquisition, Investigation, Project administration, Software, Validation, Writing – original draft, Writing – review & editing. SL: Conceptualization, Data curation, Formal Analysis, Funding acquisition, Investigation, Methodology, Supervision, Visualization, Writing – original draft, Writing – review & editing. NS: Conceptualization, Data curation, Project administration, Writing – review & editing. YY: Data curation, Methodology, Resources, Writing – review & editing. XD: Project administration, Validation, Writing – review & editing. BH: Data curation, Formal Analysis, Project administration, Writing – review & editing. CS: Conceptualization, Methodology, Writing – review & editing. CW: Data curation, Formal Analysis, Writing – review & editing. HL: Data curation, Project administration, Writing – original draft. DC: Investigation, Software, Supervision, Writing – review & editing. CH: Formal Analysis, Resources, Validation, Visualization, Writing – review & editing. PH: Formal Analysis, Project administration, Writing – review & editing. TZ: Writing – review & editing.
