# Supplementary material for: Gut microbiota composition is associated with disease severity and host immune responses in COVID-19
Source: Front Cell Infect Microbiol. 2023 Dec 12;13:1274690. doi: 10.3389/fcimb.2023.1274690 (PMC10749918; doi:10.3389/fcimb.2023.1274690)
Supplement: Supplementary file 1 [file DataSheet_1.pdf]

Table S1. The associations between microbial species and clinical traits.

			WBC		Neutrophils		Neutrophils(%)	
			**p-value**	**r**	**p-value**	**r**	**p-value**	**r**
phylum	1	Firmicutes	0.687711	-0.06557	0.722314	-0.05798	0.809407	0.039369
	2	Bacteroidetes	0.956539	0.008899	0.959114	-0.00837	0.611397	-0.08282
	3	Proteobacteria	0.232279	0.193209	0.162915	0.224915	0.28559	0.173051
	4	unidentified_Bacteria	0.157096	-0.22799	0.187627	-0.21269	0.383449	-0.1416
	5	Tenericutes	0.124478	-0.24695	0.14302	-0.23578	0.168267	-0.22216
	6	Actinobacteria	0.063314	0.296377	0.043866	0.320363	0.211971	0.201712
	7	Synergistetes	0.860439	0.028703	0.678886	0.067523	0.315665	0.162752
	8	Verrucomicrobia	0.777574	-0.0461	0.543877	-0.09887	0.270983	-0.17831
	9	Acidobacteria	0.037415	0.330244	0.084953	0.275808	0.930798	-0.01418
	10	Cyanobacteria	0.047377	0.315472	0.142358	0.236157	0.547574	-0.09797
class	1	Bacilli	0.852463	0.030362	0.522233	0.104207	0.031954	0.339764
	2	Clostridia	0.376917	-0.14353	0.182854	-0.21496	0.014734	-0.38293
	3	Bacteroidia	0.957364	0.00873	0.958383	-0.00852	0.611324	-0.08284
	4	Negativicutes	0.437768	0.126201	0.72463	0.057476	0.350112	-0.15169
	5	Gammaproteobacteria	0.298152	0.168669	0.206855	0.203942	0.282949	0.173988
	6	unidentified_Bacteria	0.157096	-0.22799	0.187627	-0.21269	0.383449	-0.1416
	7	Mollicutes	0.124478	-0.24695	0.14302	-0.23578	0.168267	-0.22216
	8	unidentified_Actinobacteria	0.056861	0.303586	0.038302	0.328807	0.253863	0.18471
	9	Erysipelotrichia	0.692465	-0.06452	0.521069	-0.1045	0.271598	-0.17808
	10	Synergistia	0.860439	0.028703	0.678886	0.067523	0.315665	0.162752
order	1	Lactobacillales	0.852152	0.030426	0.522739	0.104081	0.031964	0.339746
	2	Clostridiales	0.377019	-0.1435	0.182904	-0.21493	0.014737	-0.38292
	3	Bacteroidales	0.990947	0.001853	0.933421	-0.01364	0.620687	-0.08068
	4	Selenomonadales	0.437768	0.126201	0.72463	0.057476	0.350112	-0.15169
	5	Enterobacteriales	0.933199	0.013687	0.762973	0.049216	0.429423	0.128494

	6	unidentified_Gammaproteobacteria	0.153361	0.230003	0.129482	0.243821	0.473387	0.116676
	7	Campylobacterales	0.144385	-0.235	0.175417	-0.21857	0.385426	-0.14102
	8	Erysipelotrichales	0.692465	-0.06452	0.521069	-0.1045	0.271598	-0.17808
	9	Actinomycetales	0.285877	0.172949	0.222628	0.197184	0.368951	0.14592
	10	Synergistales	0.860439	0.028703	0.678886	0.067523	0.315665	0.162752
family	1	Enterococcaceae	0.942281	-0.01182	0.679617	0.067361	0.036823	0.331219
	2	Lachnospiraceae	0.308659	-0.16509	0.133309	-0.24149	0.010757	-0.39902
	3	Lactobacillaceae	0.419883	0.131145	0.398598	0.137186	0.487997	0.11288
	4	Tannerellaceae	0.551246	-0.09707	0.625381	-0.0796	0.653236	0.073264
	5	Bacteroidaceae	0.815079	-0.03818	0.758715	-0.05013	0.332158	-0.15737
	6	Prevotellaceae	0.356741	0.149639	0.429361	0.128511	0.792984	0.042834
	7	Ruminococcaceae	0.779734	0.045643	0.921002	0.016193	0.616498	-0.08164
	8	Veillonellaceae	0.277211	0.176044	0.586505	0.08864	0.247267	-0.18725
	9	Enterobacteriaceae	0.933199	0.013687	0.762973	0.049216	0.429423	0.128494
	10	Burkholderiaceae	0.173279	0.219636	0.143666	0.235407	0.462197	0.119626
genus	1	Enterococcus	0.942281	-0.01182	0.679617	0.067361	0.036823	0.331219
	2	Blautia	0.777768	-0.04606	0.507934	-0.10779	0.181225	-0.21574
	3	Lactobacillus	0.419883	0.131145	0.398598	0.137186	0.487997	0.11288
	4	Parabacteroides	0.551246	-0.09707	0.625381	-0.0796	0.653236	0.073264
	5	Bacteroides	0.815079	-0.03818	0.758715	-0.05013	0.332158	-0.15737
	6	unidentified_Prevotellaceae	0.369623	0.145718	0.446938	0.12371	0.80997	0.039251
	7	Megamonas	0.77485	0.046682	0.758378	-0.0502	0.240994	-0.18972
	8	Enterobacter	0.773945	0.046875	0.604855	0.084342	0.339185	0.155123
	9	unidentified_Ruminococcaceae	0.632388	0.077994	0.459884	0.12024	0.423401	0.130163
	10	Faecalibacterium	0.72556	0.057274	0.620182	-0.08079	0.029244	-0.345
	11	Subdoligranulum	0.828491	0.035364	0.589445	0.087948	0.175882	0.218345
	12	unidentified_Lachnospiraceae	0.675782	-0.06821	0.789626	-0.04355	0.928314	-0.01469
	13	Achromobacter	0.273989	0.17721	0.217262	0.199445	0.40277	0.135987

	14	Dialister	0.012468	0.391564	0.025881	0.352077	0.91738	0.016938
	15	Phascolarctobacterium	0.577269	-0.09083	0.724743	-0.05745	0.699787	0.062904
	16	Veillonella	0.125076	-0.24657	0.179564	-0.21654	0.541984	-0.09933
	17	Lachnospira	0.343651	-0.15371	0.193489	-0.20996	0.01461	-0.38337
	18	Streptococcus	0.304965	-0.16634	0.188288	-0.21238	0.104088	-0.26079
	19	Fusicatenibacter	0.154288	-0.2295	0.154364	-0.22946	0.11044	-0.25628
	20	Campylobacter	0.144385	-0.235	0.175417	-0.21857	0.385426	-0.14102
	21	Butyricicoccus	0.178703	-0.21696	0.171901	-0.22032	0.1346	-0.24071
	22	Alistipes	0.30657	0.165798	0.371079	0.14528	0.833232	0.034372
	23	Romboutsia	0.251605	-0.18558	0.121411	-0.24892	0.003416	-0.45195
	24	Sellimonas	0.519428	0.104906	0.504055	0.108775	0.857086	0.0294
	25	Agathobacter	0.152278	-0.23059	0.125824	-0.2461	0.188613	-0.21222
	26	Terrisporobacter	0.223341	-0.19689	0.174899	-0.21883	0.09322	-0.26903
	27	unidentified_Clostridiales	0.066289	-0.29325	0.074408	-0.28524	0.086009	-0.27491
	28	unidentified_Erysipelotrichaceae	0.927949	-0.01476	0.724396	-0.05753	0.429992	-0.12834
	29	Anaerostipes	0.84627	0.031651	0.660773	0.071568	0.532706	0.10161
	30	Actinomyces	0.285877	0.172949	0.222628	0.197184	0.368951	0.14592
	1	Enterococcus_faecium	0.868606	0.027008	0.508195	0.107727	0.022773	0.359335
	2	Parabacteroides_merdae	0.379695	-0.14271	0.479739	-0.11502	0.70247	0.062315
	3	Lactobacillus_salivarius	0.898515	-0.02082	0.984694	-0.00313	0.678831	0.067535
	4	Bacteroides_thetaiotaomicron	0.384842	-0.14119	0.319693	-0.16142	0.203165	-0.20557
	5	Bacteroides_dorei	0.191684	-0.21079	0.233122	-0.19287	0.488954	-0.11263
species	6	Bacteroides_ovatus	0.654487	-0.07298	0.712553	-0.06011	0.923741	-0.01563
	7	Prevotella_copri	0.426527	0.129295	0.455828	0.121322	0.696922	0.063535
	8	Bacteroides_vulgatus	0.926586	-0.01504	0.836137	0.033765	0.294686	0.169866
	9	Enterobacter_cloacae	0.77402	0.046859	0.604939	0.084322	0.33925	0.155102
	10	Bacteroides_plebeius	0.706634	0.061402	0.839781	0.033004	0.348327	-0.15225
	11	Clostridiales_bacterium_24-4c	0.595485	0.086529	0.455676	0.121362	0.338054	0.155482

12	Enterococcus_avium	0.252319	-0.1853	0.204165	-0.20513	0.144973	-0.23466
13	Clostridium_sp_Marseille-P3244	0.193508	-0.20995	0.233991	-0.19252	0.641892	-0.07583
14	Phascolarctobacterium_faecium	0.576598	-0.09099	0.721394	-0.05818	0.711834	0.060264
15	Bacteroides_uniformis	0.050885	0.31087	0.129024	0.244104	0.746412	-0.05277
16	Bacteroides_coprocola	0.006692	0.421927	0.030164	0.343176	0.717491	-0.05903
17	Parabacteroides_distasonis	0.265666	0.180266	0.42901	0.128608	0.91448	0.017534
18	Lactobacillus_reuteri	0.209081	0.202968	0.482317	0.114349	0.560969	-0.09472
19	Campylobacter_coli	0.146037	-0.23406	0.178079	-0.21727	0.393345	-0.1387
20	Bacteroides_fragilis	0.48396	-0.11392	0.527033	-0.10301	0.649333	-0.07414
21	Blautia_massiliensis	0.989312	0.002187	0.937353	-0.01283	0.961661	0.007849
22	Lactobacillus_mucosae	0.223697	0.196738	0.506869	0.108062	0.526668	-0.1031
23	Streptococcus_salivarius_subsp_thermophilus	0.395273	-0.13815	0.238182	-0.19083	0.031728	-0.34019
24	Okadaella_gastrococcus	0.206843	-0.20395	0.178704	-0.21696	0.234746	-0.19221
25	[Eubacterium]_hallii	0.918754	0.016655	0.953779	0.009464	0.789837	-0.0435
26	Enterococcus_faecalis	0.647841	0.074482	0.472165	0.116996	0.273781	0.177286
27	Coprococcus_eutactus	0.002169	0.470818	0.001511	0.485107	0.360349	0.148532
28	Ruminococcus_bromii	0.800164	0.041317	0.717381	0.059054	0.879616	0.024728
29	Lactobacillus_fermentum	0.515703	0.105838	0.794974	0.042413	0.660874	-0.07155
30	Eubacterium_ramulus	0.150544	-0.23155	0.141502	-0.23665	0.152573	-0.23043

			Lymphocytes		Lymphocytes(%)		CRP	
			**p-value**	**r**	**p-value**	**r**	**p-value**	**r**
phylum	1	Firmicutes	0.908364	-0.01879	0.807806	-0.03971	0.946904	-0.01118
	2	Bacteroidetes	0.735796	0.055057	0.546703	0.098179	0.760522	-0.05112
	3	Proteobacteria	0.508391	-0.10768	0.241649	-0.18946	0.553131	0.099288
	4	unidentified_Bacteria	0.620977	-0.08061	0.551542	0.097002	0.918397	-0.01719
	5	Tenericutes	0.689826	-0.0651	0.305219	0.166255	0.520575	-0.10751
	6	Actinobacteria	0.40291	-0.13595	0.149802	-0.23196	0.029275	0.353894
	7	Synergistetes	0.463472	-0.11929	0.40548	-0.13521	1.22E-08	0.773777
	8	Verrucomicrobia	0.290154	0.171445	0.521505	0.104388	0.785248	-0.04571
	9	Acidobacteria	0.19795	0.207918	0.727199	-0.05692	0.376077	0.147735
	10	Cyanobacteria	0.065437	0.294133	0.953889	0.009442	0.645955	0.076984
class	1	Bacilli	0.12857	-0.24439	0.027442	-0.3487	0.674332	0.070435
	2	Clostridia	0.181954	0.215387	0.034009	0.336036	0.798999	-0.04272
	3	Bacteroidia	0.736196	0.054971	0.546588	0.098207	0.760486	-0.05113
	4	Negativicutes	0.056613	0.303875	0.035947	0.332685	0.187675	-0.21843
	5	Gammaproteobacteria	0.458427	-0.12063	0.253348	-0.18491	0.586134	0.091183
	6	unidentified_Bacteria	0.620977	-0.08061	0.551542	0.097002	0.918397	-0.01719
	7	Mollicutes	0.689826	-0.0651	0.305219	0.166255	0.520575	-0.10751
	8	unidentified_Actinobacteria	0.455975	-0.12128	0.231048	-0.19371	0.391129	0.143181
	9	Erysipelotrichia	0.447429	0.123578	0.715299	0.059508	0.101658	0.269598
	10	Synergistia	0.463472	-0.11929	0.40548	-0.13521	1.22E-08	0.773777
order	1	Lactobacillales	0.130579	-0.24315	0.027511	-0.34855	0.682295	0.068616
	2	Clostridiales	0.181908	0.21541	0.034013	0.336029	0.799069	-0.0427
	3	Bacteroidales	0.764876	0.048809	0.546277	0.098283	0.742504	-0.0551
	4	Selenomonadales	0.056613	0.303875	0.035947	0.332685	0.187675	-0.21843
	5	Enterobacteriales	0.620268	-0.08077	0.531539	-0.1019	0.835806	-0.03477
	6	unidentified_Gammaproteobacteria	0.541408	-0.09947	0.322599	-0.16047	0.275859	0.181355

	7	Campylobacterales	0.602366	-0.08492	0.548795	0.09767	0.363849	-0.15151
	8	Erysipelotrichales	0.447429	0.123578	0.715299	0.059508	0.101658	0.269598
	9	Actinomycetales	0.383432	-0.1416	0.292184	-0.17074	0.369259	0.149833
	10	Synergistales	0.463472	-0.11929	0.40548	-0.13521	1.22E-08	0.773777
family	1	Enterococcaceae	0.110655	-0.25613	0.041742	-0.32348	0.791715	0.044299
	2	Lachnospiraceae	0.206273	0.204199	0.00738	0.417326	0.938779	-0.01289
	3	Lactobacillaceae	0.827596	-0.03555	0.390719	-0.13947	0.653828	0.075156
	4	Tannerellaceae	0.541573	-0.09943	0.545334	-0.09851	0.917178	-0.01745
	5	Bacteroidaceae	0.763945	0.049008	0.185802	0.213549	0.802603	-0.04194
	6	Prevotellaceae	0.480155	0.11491	0.749881	-0.05202	0.648204	-0.07646
	7	Ruminococcaceae	0.382706	0.141819	0.957389	0.008725	0.592985	-0.08953
	8	Veillonellaceae	0.022845	0.359157	0.576423	0.091028	0.582211	-0.09214
	9	Enterobacteriaceae	0.620268	-0.08077	0.531539	-0.1019	0.835806	-0.03477
	10	Burkholderiaceae	0.517206	-0.10546	0.329238	-0.15831	0.295889	0.174085
genus	1	Enterococcus	0.110655	-0.25613	0.041742	-0.32348	0.791715	0.044299
	2	Blautia	0.334914	0.156484	0.171456	0.220547	0.893635	-0.02244
	3	Lactobacillus	0.827596	-0.03555	0.390719	-0.13947	0.653828	0.075156
	4	Parabacteroides	0.541573	-0.09943	0.545334	-0.09851	0.917178	-0.01745
	5	Bacteroides	0.763945	0.049008	0.185802	0.213549	0.802603	-0.04194
	6	unidentified_Prevotellaceae	0.47983	0.114994	0.765383	-0.0487	0.690907	-0.06666
	7	Megamonas	0.029258	0.344967	0.496215	0.110771	0.534844	-0.10388
	8	Enterobacter	0.624575	-0.07978	0.497501	-0.11044	0.676199	-0.07001
	9	unidentified_Ruminococcaceae	0.58368	-0.08931	0.4812	-0.11464	0.538987	0.10283
	10	Faecalibacterium	0.000796	0.50903	0.205962	0.204336	0.420369	-0.13462
	11	Subdoligranulum	0.443508	-0.12464	0.249772	-0.18628	0.521601	-0.10725
	12	unidentified_Lachnospiraceae	0.595451	-0.08654	0.929039	-0.01454	0.024602	0.364152
	13	Achromobacter	0.423634	-0.1301	0.331739	-0.1575	0.362001	0.152092
	14	Dialister	0.163243	0.224745	0.871385	-0.02643	0.594151	-0.08924

	15	Phascolarctobacterium	0.557014	-0.09568	0.002238	0.469543	0.133679	-0.24776
	16	Veillonella	0.460613	-0.12005	0.811806	0.038865	0.404476	0.139227
	17	Lachnospira	0.241951	0.189337	0.006383	0.424124	0.287991	-0.17691
	18	Streptococcus	0.294186	0.170039	0.201341	0.20639	0.702563	-0.06402
	19	Fusicatenibacter	0.792888	-0.04285	0.393275	0.138725	0.95068	-0.01038
	20	Campylobacter	0.602366	-0.08492	0.548795	0.09767	0.363849	-0.15151
	21	Butyricicoccus	0.866971	-0.02735	0.19734	0.208195	0.955198	0.009428
	22	Alistipes	0.80556	-0.04018	0.376896	-0.14354	0.106104	0.26628
	23	Romboutsia	0.256552	0.183686	0.044808	0.319021	0.377615	-0.14726
	24	Sellimonas	0.593684	-0.08695	0.540509	-0.09969	0.688965	0.067099
	25	Agathobacter	0.65899	0.071968	0.083492	0.277059	0.220306	-0.20355
	26	Terrisporobacter	0.892785	0.022006	0.376159	0.14376	0.373867	-0.14841
	27	unidentified_Clostridiales	0.662703	-0.07113	0.281326	0.174567	0.963268	0.007729
	28	unidentified_Erysipelotrichaceae	0.396762	0.137715	0.806008	0.040085	0.051025	0.318872
	29	Anaerostipes	0.729874	-0.05634	0.001559	0.483889	0.298386	-0.1732
	30	Actinomyces	0.383432	-0.1416	0.292184	-0.17074	0.369259	0.149833
	1	Enterococcus_faecium	0.129822	-0.24361	0.03936	-0.32713	0.888002	0.023632
	2	Parabacteroides_merdae	0.514321	-0.10618	0.710961	-0.06045	0.833214	-0.03533
	3	Lactobacillus_salivarius	0.736006	-0.05501	0.655395	-0.07278	0.736258	-0.05648
	4	Bacteroides_thetaiotaomicron	0.892049	0.022158	0.542781	0.099136	0.497728	-0.11343
	5	Bacteroides_dorei	0.648204	-0.0744	0.651777	0.073593	0.357116	-0.15363
species	6	Bacteroides_ovatus	0.777407	-0.04614	0.980706	0.003949	0.177537	0.223423
	7	Prevotella_copri	0.838205	0.033333	0.622935	-0.08016	0.937353	-0.01319
	8	Bacteroides_vulgatus	0.318718	-0.16174	0.951921	-0.00985	0.922336	-0.01636
	9	Enterobacter_cloacae	0.624603	-0.07978	0.497534	-0.11043	0.676401	-0.06996
	10	Bacteroides_plebeius	0.394481	0.138375	0.130808	0.243006	0.379634	-0.14665
	11	Clostridiales_bacterium_24-4c	0.622564	-0.08025	0.459694	-0.12029	0.50723	0.110952
	12	Enterococcus_avium	0.948852	0.010475	0.471772	0.117099	0.614497	-0.08438

13	Clostridium_sp_Marseille-P3244	0.540109	-0.09979	0.914991	0.017429	0.376206	0.147695
14	Phascolarctobacterium_faecium	0.564108	-0.09397	0.002155	0.471069	0.111914	-0.2621
15	Bacteroides_uniformis	0.111405	0.255607	0.821069	0.036919	0.29572	0.174145
16	Bacteroides_coprocola	0.018258	0.371518	0.759565	0.049945	0.63053	-0.08059
17	Parabacteroides_distasonis	0.808188	0.039626	0.366885	-0.14654	0.729324	0.058024
18	Lactobacillus_reuteri	0.030458	0.342605	0.713655	0.059867	0.292599	0.175256
19	Campylobacter_coli	0.597636	-0.08603	0.56473	0.093821	0.2123	0.2254
20	Bacteroides_fragilis	0.682926	-0.06663	0.984991	0.003072	0.863456	0.028856
21	Blautia_massiliensis	0.841203	0.032708	0.000563	0.521274	0.496711	-0.1137
22	Lactobacillus_mucosae	0.03014	0.343224	0.687229	0.065673	0.305498	0.170709
23	Streptococcus_salivarius_subsp_thermophilus	0.302908	0.167041	0.075594	0.284131	0.10896	0.264204
24	Okadaella_gastrococcus	0.771437	0.047409	0.447874	0.123457	0.144861	-0.24106
25	[Eubacterium]_hallii	0.751801	0.051609	0.97225	-0.00568	0.018449	0.380478
26	Enterococcus_faecalis	0.292919	-0.17048	0.258274	-0.18303	2.98E-08	0.760742
27	Coprococcus_eutactus	0.948418	0.010564	0.416627	-0.13206	0.680315	0.069068
28	Ruminococcus_bromii	0.976094	-0.00489	0.824874	-0.03612	0.090259	0.278644
29	Lactobacillus_fermentum	0.13998	0.237533	0.872989	0.0261	0.656797	0.074469
30	Eubacterium_ramulus	0.834372	-0.03413	0.391091	0.13936	0.834119	-0.03514

			ALB		PCT		IL-6	
			p-value	r	p-value	r	p-value	r
phylum	1	Firmicutes	0.607721	0.083675	0.165226	-0.22668	0.253469	0.187331
	2	Bacteroidetes	0.647078	-0.07465	0.208896	0.205751	0.202504	-0.20861
	3	Proteobacteria	0.873364	-0.02602	0.986016	0.002901	0.942251	0.011989
	4	unidentified_Bacteria	0.644437	-0.07525	0.260281	0.184714	0.661171	-0.07245
	5	Tenericutes	0.482442	0.114316	0.293145	-0.17268	0.55622	-0.09717
	6	Actinobacteria	0.697069	-0.0635	0.603867	0.085718	0.789806	0.044098
	7	Synergistetes	0.961865	0.007808	0.102218	0.265598	0.456374	0.122812
	8	Verrucomicrobia	0.332734	-0.15718	0.502812	-0.11056	0.761346	-0.05024
	9	Acidobacteria	0.590586	-0.08768	0.0618	0.301878	0.146877	0.236676
	10	Cyanobacteria	0.819109	0.03733	0.027548	0.352911	0.260088	0.184787
class	1	Bacilli	0.558624	0.09529	0.236114	-0.19422	0.401049	0.13832
	2	Clostridia	0.471053	-0.11729	0.477648	0.117124	0.803065	0.041258
	3	Bacteroidia	0.647247	-0.07462	0.209134	0.205646	0.202278	-0.20871
	4	Negativicutes	0.269202	0.17896	0.446019	-0.12563	0.202577	-0.20858
	5	Gammaproteobacteria	0.909677	-0.01852	0.908295	-0.01906	0.9661	-0.00703
	6	unidentified_Bacteria	0.644437	-0.07525	0.260281	0.184714	0.661171	-0.07245
	7	Mollicutes	0.482442	0.114316	0.293145	-0.17268	0.55622	-0.09717
	8	unidentified_Actinobacteria	0.712033	-0.06022	0.928741	0.014801	0.907064	0.019321
	9	Erysipelotrichia	0.416159	-0.13219	0.709648	0.061567	0.19875	0.210316
	10	Synergistia	0.961865	0.007808	0.102218	0.265598	0.456374	0.122812
order	1	Lactobacillales	0.552223	0.096837	0.237727	-0.19356	0.405054	0.137158
	2	Clostridiales	0.471042	-0.11729	0.477642	0.117126	0.803081	0.041255
	3	Bacteroidales	0.649486	-0.07411	0.230381	0.196569	0.192018	-0.21344
	4	Selenomonadales	0.269202	0.17896	0.446019	-0.12563	0.202577	-0.20858
	5	Enterobacteriales	0.566834	0.093316	0.522836	-0.10546	0.632541	-0.07902
	6	unidentified_Gammaproteobacteria	0.390506	-0.13953	0.587042	0.089713	0.651965	0.074548

	7	Campylobacterales	0.643082	-0.07556	0.270782	0.180767	0.642715	-0.07667
	8	Erysipelotrichales	0.416159	-0.13219	0.709648	0.061567	0.19875	0.210316
	9	Actinomycetales	0.392643	-0.13891	0.846653	0.032	0.802369	0.041407
	10	Synergistales	0.961865	0.007808	0.102218	0.265598	0.456374	0.122812
family	1	Enterococcaceae	0.137566	0.238947	0.176505	-0.22093	0.347295	0.154619
	2	Lachnospiraceae	0.790818	-0.04329	0.554111	0.097685	0.419209	0.133104
	3	Lactobacillaceae	0.036714	-0.3314	0.69306	0.065259	0.872265	0.026607
	4	Tannerellaceae	0.328357	0.158593	0.737327	-0.05547	0.511621	-0.10831
	5	Bacteroidaceae	0.609536	-0.08325	0.376513	0.145588	0.476567	-0.11741
	6	Prevotellaceae	0.403037	-0.13591	0.297353	0.171208	0.433702	-0.12903
	7	Ruminococcaceae	0.272967	-0.17758	0.607429	0.084878	0.513158	-0.10792
	8	Veillonellaceae	0.216154	0.199916	0.850097	-0.03127	0.422537	-0.13216
	9	Enterobacteriaceae	0.566834	0.093316	0.522836	-0.10546	0.632541	-0.07902
	10	Burkholderiaceae	0.410671	-0.13374	0.638919	0.077544	0.709139	0.06168
genus	1	Enterococcus	0.137566	0.238947	0.176505	-0.22093	0.347295	0.154619
	2	Blautia	0.882367	0.024159	0.428516	0.130481	0.112721	0.258049
	3	Lactobacillus	0.036714	-0.3314	0.69306	0.065259	0.872265	0.026607
	4	Parabacteroides	0.328357	0.158593	0.737327	-0.05547	0.511621	-0.10831
	5	Bacteroides	0.609536	-0.08325	0.376513	0.145588	0.476567	-0.11741
	6	unidentified_Prevotellaceae	0.463428	-0.1193	0.266713	0.182284	0.452179	-0.12395
	7	Megamonas	0.519151	0.104975	0.775541	-0.04717	0.480481	-0.11638
	8	Enterobacter	0.592552	0.087218	0.592053	-0.08852	0.557979	-0.09674
	9	unidentified_Ruminococcaceae	0.307993	-0.16532	0.429025	0.130338	0.366979	0.148487
	10	Faecalibacterium	0.258871	0.182808	0.904682	0.019818	0.230868	-0.19637
	11	Subdoligranulum	0.01646	-0.37708	0.507909	0.109257	0.377679	-0.14524
	12	unidentified_Lachnospiraceae	0.895557	-0.02143	0.488784	0.1142	0.849425	0.031415
	13	Achromobacter	0.387178	-0.1405	0.84738	0.031847	0.785596	0.045002
	14	Dialister	0.023999	0.356378	0.49385	-0.11288	0.349887	-0.1538

	15	Phascolarctobacterium	0.833577	-0.0343	0.260119	-0.18478	0.349868	-0.15381
	16	Veillonella	0.123116	-0.24782	0.270419	0.180902	0.416594	0.133847
	17	Lachnospira	0.990112	-0.00202	0.450424	-0.12443	0.290806	-0.17351
	18	Streptococcus	0.38242	0.141903	0.451968	-0.12401	0.149579	-0.23515
	19	Fusicatenibacter	0.552035	-0.09688	0.860867	0.029003	0.554338	-0.09763
	20	Campylobacter	0.643082	-0.07556	0.270782	0.180767	0.642715	-0.07667
	21	Butyricicoccus	0.247925	-0.187	0.898281	0.021156	0.764142	-0.04963
	22	Alistipes	0.328107	-0.15867	0.3852	0.142984	0.321031	0.163142
	23	Romboutsia	0.739155	0.054332	0.576778	-0.09218	0.450951	-0.12428
	24	Sellimonas	0.144119	-0.23515	0.347061	0.154693	0.065576	0.297805
	25	Agathobacter	0.831538	-0.03473	0.225512	-0.1986	0.09107	-0.27429
	26	Terrisporobacter	0.74453	0.053173	0.659806	-0.07276	0.458328	-0.12228
	27	unidentified_Clostridiales	0.830971	0.034845	0.83624	0.034203	0.487332	0.114579
	28	unidentified_Erysipelotrichaceae	0.999922	1.59E-05	0.605747	0.085274	0.198184	0.210576
	29	Anaerostipes	0.10817	0.257866	0.510118	-0.10869	0.2692	-0.18136
	30	Actinomyces	0.392643	-0.13891	0.846653	0.032	0.802369	0.041407
	1	Enterococcus_faecium	0.129269	0.243953	0.205735	-0.20716	0.307655	0.16765
	2	Parabacteroides_merdae	0.264848	0.18057	0.462074	-0.12127	0.463111	-0.121
	3	Lactobacillus_salivarius	0.027858	-0.34783	0.591641	-0.08862	0.976941	-0.00478
	4	Bacteroides_thetaiotaomicron	0.919781	0.016444	0.839737	-0.03346	0.529438	-0.1038
	5	Bacteroides_dorei	0.407336	-0.13468	0.326815	0.161229	0.520438	-0.10607
	6	Bacteroides_ovatus	0.130927	-0.24293	0.527324	-0.10433	0.318039	0.16414
species	7	Prevotella_copri	0.354767	-0.15025	0.063684	0.299822	0.633546	-0.07878
	8	Bacteroides_vulgatus	0.678735	-0.06756	0.231194	0.196233	0.564492	-0.09515
	9	Enterobacter_cloacae	0.592691	0.087185	0.591977	-0.08854	0.558138	-0.0967
	10	Bacteroides_plebeius	0.005113	0.434263	0.471052	-0.11887	0.308986	-0.1672
	11	Clostridiales_bacterium_24-4c	0.135247	-0.24032	0.6818	0.067784	0.130046	0.246683
	12	Enterococcus_avium	0.921727	0.016044	0.702632	-0.06312	0.618283	-0.08233

13	Clostridium_sp_Marseille-P3244	0.567814	-0.09308	0.777966	-0.04664	0.475701	0.117639
14	Phascolarctobacterium_faecium	0.83201	-0.03463	0.247606	-0.18962	0.343659	-0.15577
15	Bacteroides_uniformis	0.932879	-0.01375	0.09939	0.267731	0.200549	0.209495
16	Bacteroides_coprocola	0.233301	0.192795	0.601985	-0.08616	0.661871	-0.07229
17	Parabacteroides_distasonis	0.579604	-0.09027	0.052079	0.313357	0.704653	0.062675
18	Lactobacillus_reuteri	0.499576	0.109913	0.000621	0.523829	0.71354	0.060705
19	Campylobacter_coli	0.640302	-0.07619	0.268737	0.181528	0.64711	-0.07566
20	Bacteroides_fragilis	0.008535	-0.41038	0.986508	0.002799	0.975695	0.005043
21	Blautia_massiliensis	0.462037	0.119668	0.740633	-0.05475	0.585345	-0.09012
22	Lactobacillus_mucosae	0.464083	0.119126	0.000726	0.518243	0.747868	0.053167
23	Streptococcus_salivarius_subsp_thermophilus	0.273825	0.17727	0.446895	0.125391	0.634345	-0.0786
24	Okadaella_gastrococcus	0.486144	0.113358	0.175527	-0.22142	0.137389	-0.24221
25	[Eubacterium]_hallii	0.563713	0.094065	0.305906	0.168249	0.578389	-0.09179
26	Enterococcus_faecalis	0.721754	-0.0581	0.146908	0.236658	0.403703	0.137549
27	Coprococcus_eutactus	0.066181	0.293361	0.89649	0.02153	0.96875	-0.00648
28	Ruminococcus_bromii	0.538153	0.10027	0.067797	0.295496	0.387523	-0.14229
29	Lactobacillus_fermentum	0.333977	0.156783	0.024946	0.358676	0.871786	-0.02671
30	Eubacterium_ramulus	0.142686	-0.23597	0.915656	-0.01753	0.443013	-0.12646
