# Supplementary material for: Gut microbiota composition is associated with disease severity and host immune responses in COVID-19
Source: Front Cell Infect Microbiol. 2023 Dec 12;13:1274690. doi: 10.3389/fcimb.2023.1274690 (PMC10749918; doi:10.3389/fcimb.2023.1274690)
Supplement: Supplementary file 2 [file DataSheet_2.pdf]

Gut microbiome composition is associated with disease severity and host immune responses in COVID-19

Supplementary Figures

Figure S1

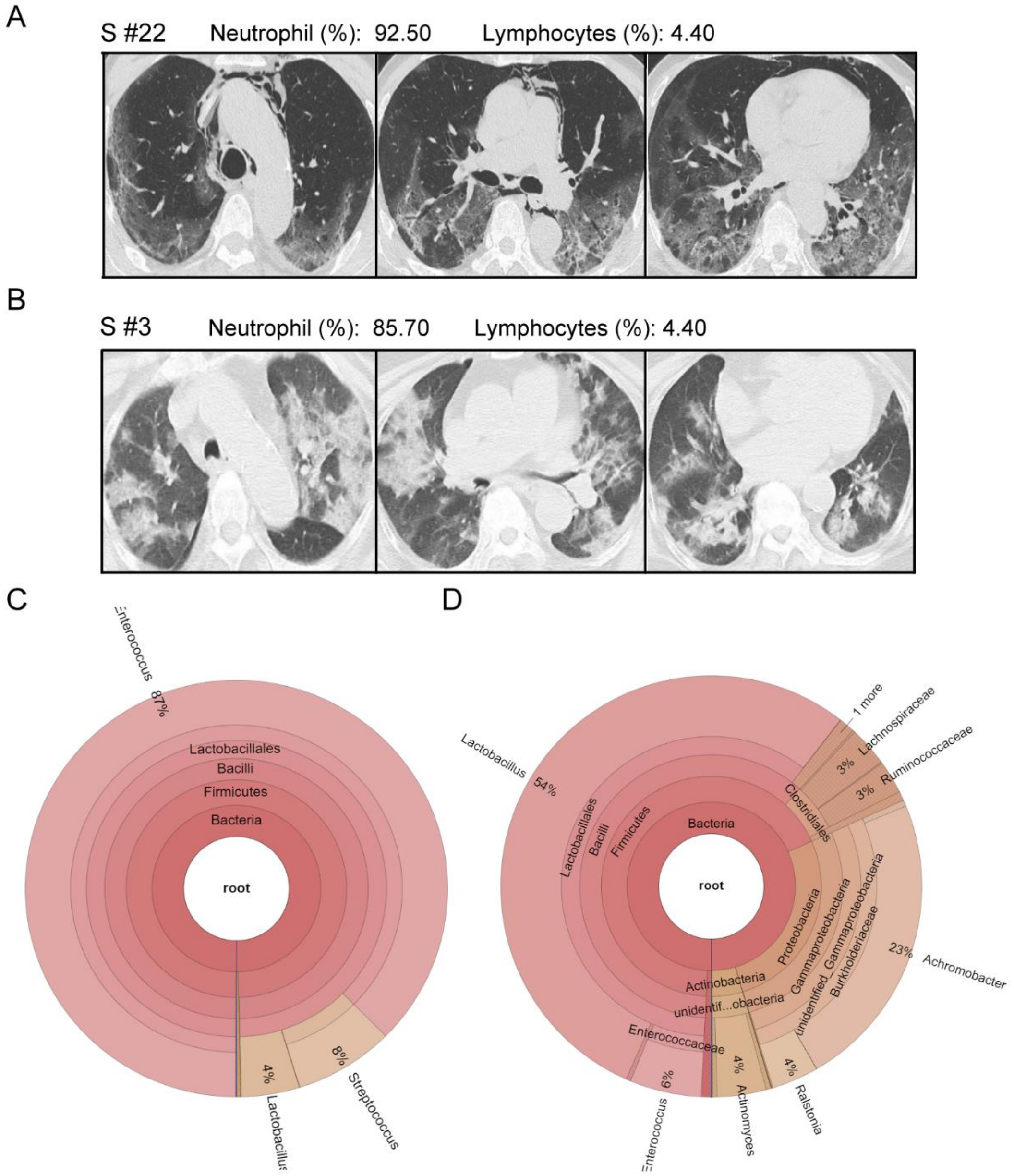

Fig. S1. Gut microbiota composition and Chest CT scan features in dead patients with COVID-19. (A, B) Chest computed tomography from severe patients that ended in death showed confluent and predominantly patchy ground glass opacities with pronounced peripheral distribution, and partial consolidation. (C, D) Species annotation was performed based on the sequence information of the OTUs, a Krona pie chart was established at the genus level. And the absolute abundance of *Enterococcus* and *Lactobacillales* accounted for 87% and 54%, respectively.

Figure S2

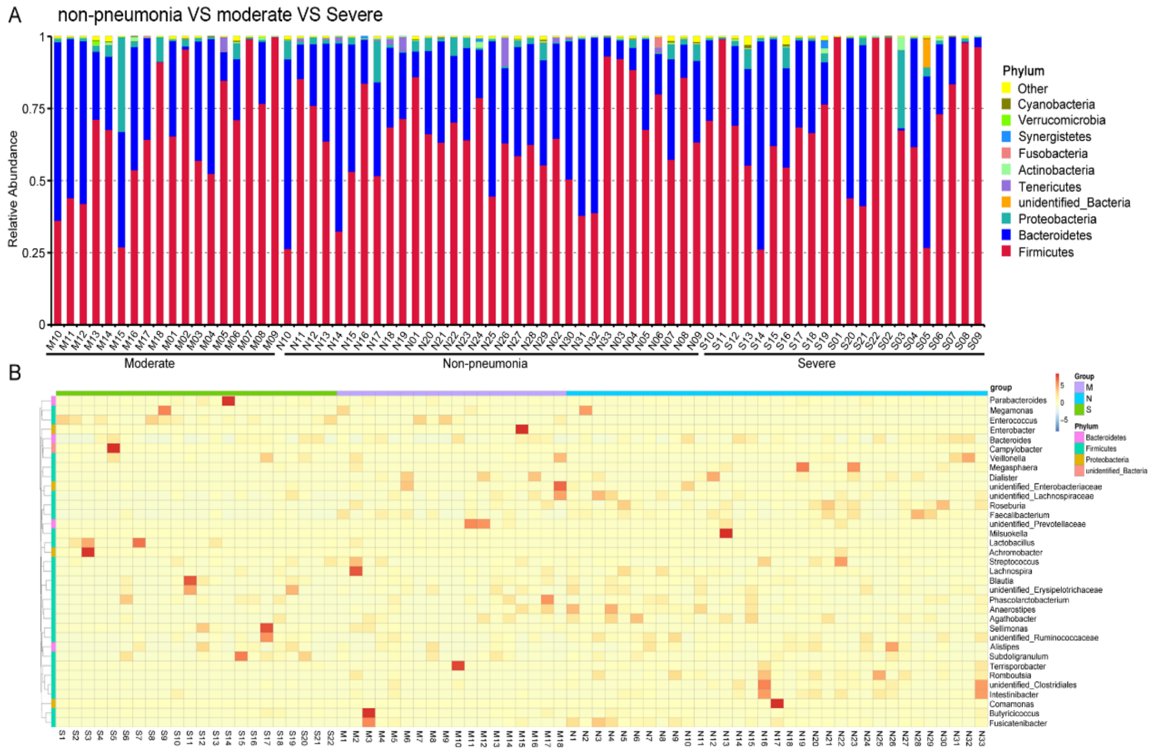

Fig. S2. Alterations in gut microbiome composition of COVID-19 patients. (A) The phylum distribution of the gut microbiota of patients with moderate, severe COVID-19 and non-pneumonia individuals. (B) Distribution of predominant gut microbes in different individuals at the genus levels.

Figure S3

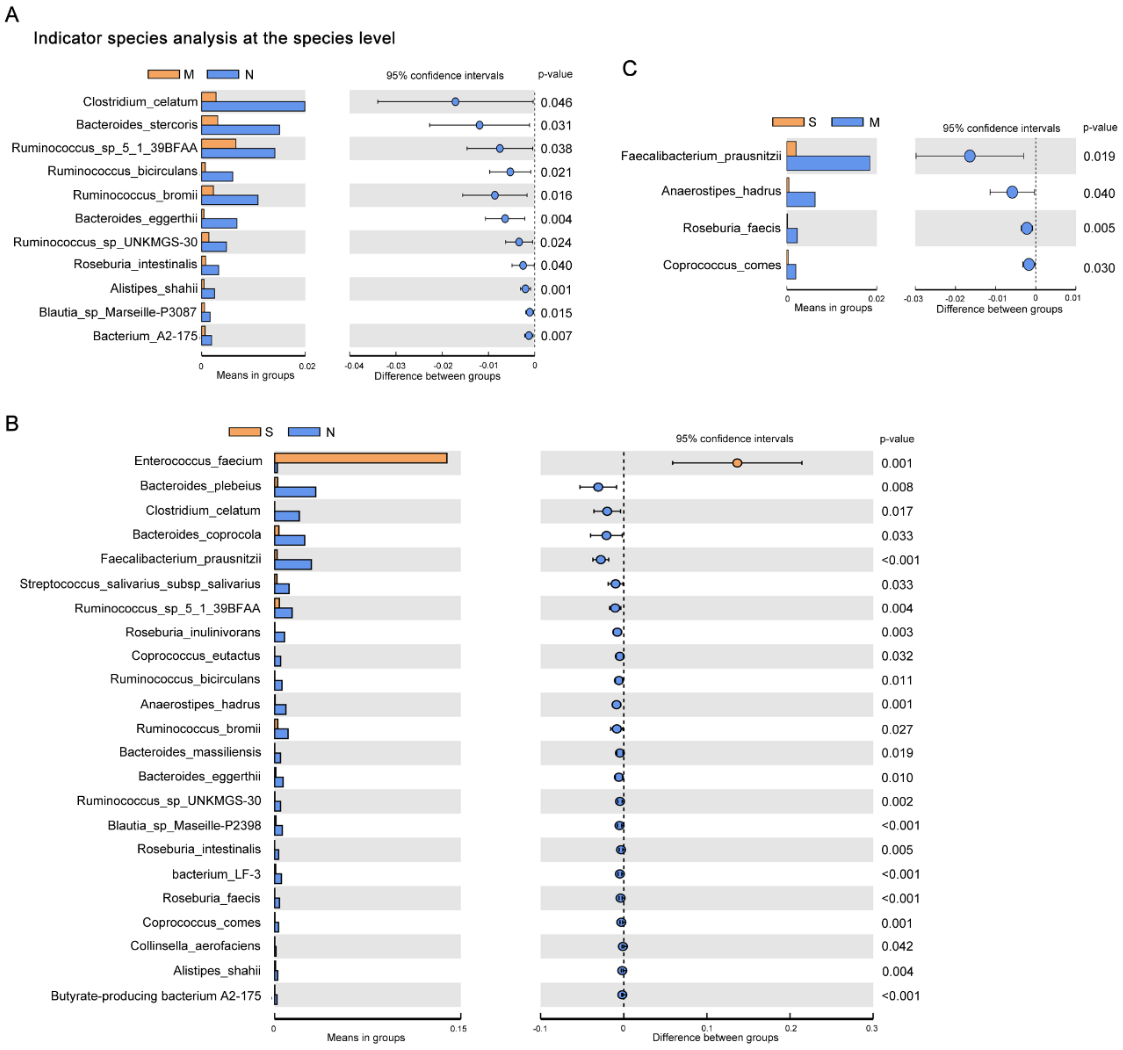

Fig. S3. Changes in the gut microbiota composition in the patients with COVID-19. Differentially bacterial species among the patients with severe, moderate COVID-19 and non-pneumonia individuals. OTUs and taxa differences are shown with p-values less than 0.05. (A) The differences between moderate group and non-pneumonia group. (B) The differences between severe group and non-pneumonia group. (C) The differences between moderate group and severe group.

Figure S4

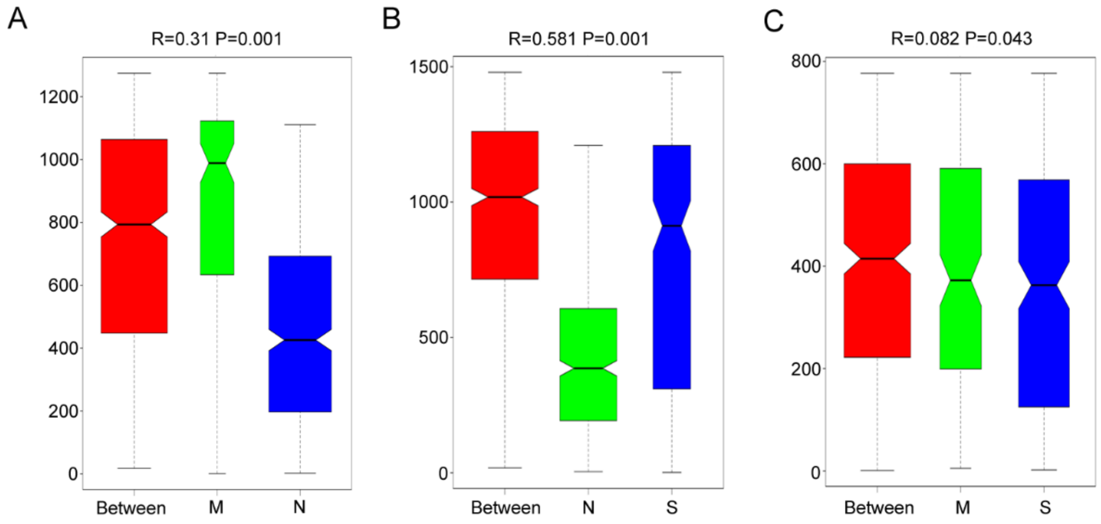

Fig. S4. Changes in bacterial microbiome diversity in COVID-19 patients. Based on the distance index ranking, ANOSIM (analysis of similarities) confirmed that the distance between groups was significantly greater than the distance within groups, indicating that the microbiome structure of different groups was significantly different. (A) Moderate vs Non-pneumonia; (B) Non-pneumonia vs Severe; (C) Moderate vs Severe.

Figure S5

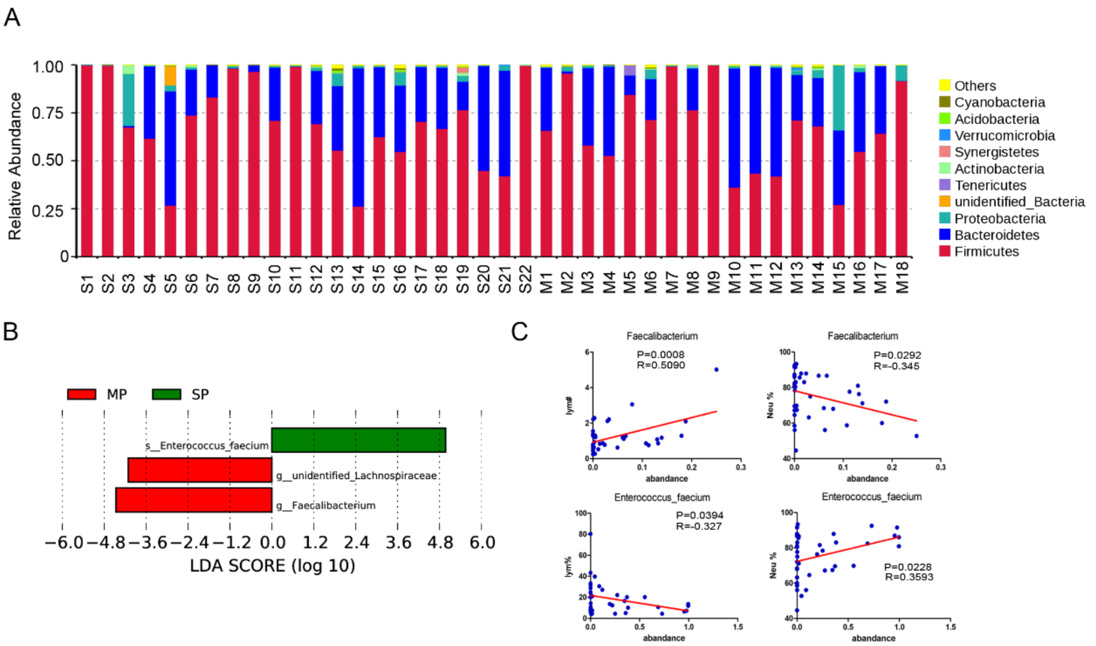

Fig. S5. Correlations between illness severity-related gut microbes and clinical parameter of COVID-19 patients

(A) Gut microbiota composition in moderate and severe COVID-19 patients. Average relative abundance of microbial phyla detected in stool samples from patients with severe and moderate COVID-19. The *Firmicutes* and *Bacteroidetes* were dominant communities in both groups. (B) The bar graph showed the LDA scores calculated for characteristics at the OTUs levels. Green and red bars referred to the severe and moderate group, respectively. (C) Analysis of correlation between illness severity-related gut microbes (*Faecalibacterium* and *Enterococcus faecium*) and the blood features. The Pearson correlation coefficient and *P* value are used for plotting.
